# Application of a Zwitterionic Hydrophobic Associating Polymer with High Salt and Heat Tolerance in Brine-Based Fracturing Fluid

**DOI:** 10.3390/polym11122005

**Published:** 2019-12-03

**Authors:** Jizhen Tian, Jincheng Mao, Wenlong Zhang, Xiaojiang Yang, Chong Lin, Meng Cun, Jinhua Mao, Jinzhou Zhao

**Affiliations:** State Key Laboratory of Oil and Gas Reservoir Geology and Exploitation, Southwest Petroleum University, Chengdu 610500, China; 201822000470@stu.swpu.edu.cn (J.T.); linc0616@163.com (C.L.); 201921000690@stu.swpu.edu.cn (M.C.); 201921000706@stu.swpu.edu.cn (J.M.);

**Keywords:** zwitterionic hydrophobic associating polymer, salt effect, fracturing fluid system, high-salinity water, oilfield wastewater

## Abstract

ZID_16_PM, a zwitterionic hydrophobic associating polymer, has equivalent positive and negative charges and some hydrophobic monomers with twin-tailed long hydrophobic chains. It exhibits a great heat resistance and salt tolerance to the common salt in formation brine (MgCl_2_, CaCl_2_, NaCl, and KCl), which is attributed to its anti-polyelectrolyte effect and strong association force. High-salinity water (seawater or formation water) can be prepared as a fracturing fluid directly. In this paper, the formation water of the West Sichuan Gas Field is directly prepared into fracturing fluid with a concentration of 0.3% ZID_16_PM (Fluid-1), and the seawater of the Gulf of Mexico is directly prepared into fracturing fluid with a concentration of 0.3% ZID_16_PM (Fluid-2). Finally, rheological measurements, proppant suspension tests, and core matrix permeability damage rate tests for the Fluid-1 and Fluid-2 are conducted. Results show that after 120 min of shearing at 140 and 160 °C, respectively, the viscosity of Fluid-1 remains in the range of 50–85 mPa·s, and the viscosity of Fluid-2 remains in the range of 60–95 mPa·s. And the wastewater produced by an oilfield in Shaanxi, Xinjiang, and Jiangsu are also prepared into fracturing fluids with a concentration of 0.3% ZID_16_PM, the viscosity of these fracturing fluids can remain 32, 42, and 45 mPa·s, respectively, after 120 min of shearing at 160 °C. All results demonstrate that the polymer ZID_16_PM displays prominent performance in fracturing fluids.

## 1. Introduction

Hydraulic fracturing technology has developed greatly over recent decades and has been the most commonly applied reservoir stimulation method for low permeability reservoirs. The fracturing fluid used in hydraulic fracturing technology must be of good viscosity and elasticity to produce the artificial fractures and deliver the proppant to the cracks [1,2,3]. Water-soluble macromolecular polymers, such as guar gum and acrylamide polymers, have been widely applied as thickeners in fracturing fluids [4]. However, conventional guar gum and acrylamide polymers can cause serious damage to the formation by blocking the pore throat and have poor salt-tolerance and temperature-tolerance [5,6]. In the process of oil and gas field development, the reservoir temperature and salinity become higher with the increase of reservoir heterogeneity. In high temperature and high salinity reservoirs, polymers such as partially hydrolyzed polyacrylamide (HPAM) currently in use, have severe apparent viscosity loss and limit the further application of HPAM [7]. Therefore, hydrophobic association polymers with excellent temperature-tolerance, salt-tolerance, and low damage have received extensive research and attention.

**Figure 1:**
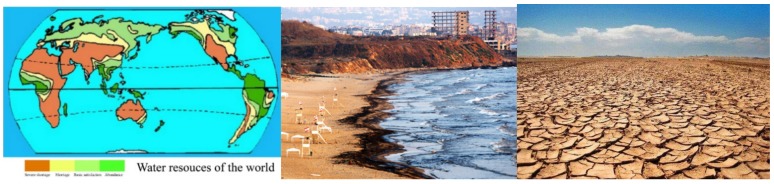
The problem of water shortage.

Water-soluble polymers with a small number of hydrophobic groups in their main chains are called hydrophobic associating polymers [8]. Due to the hydrophobic association in aqueous solution, which is caused by the interaction of intramolecular and intermolecular chains between the hydrophobic groups when they reach a certain concentration, a network structure will be formed in solution to improve the viscosity of the solution [9], so they are widely used in the oil and gas development field. This paper studies mainly the zwitterionic hydrophobic associating polymers with equal charge numbers. The molecular chains of this kind of polymer contain both anionic groups and cationic groups, and the number of the positive and negative charges are equal. Therefore, the polymer will exhibit an anti-polyelectrolyte effect, and the viscosity of the polymer solution will increase with the increase of salt concentration, which shows a strong salt-tolerance [10]. There are reports that China has entered a period of severe water shortage, and the lack of water is expected to increase from 40 billion cubic meters to 50 billion cubic meters by 2030 [11]. Although, throughout the world, China’s total freshwater resources are 2800 billion cubic meters, accounting for 6% of global water resources, ranking fourth in the world. However, the per capita fresh water resources are only 2200 cubic meters, only one-fourth of the world average level, so our country is one of the countries that are seriously lacking in freshwater resources. At present, the reuse rate of industrial water in China is only 60%, and there is almost no use of urban wastewater. The reuse of urban wastewater not only reduces pollution, but also alleviates the contradiction of water shortage [12]. As shown in Figure 1, at the same time, there are many areas of the world that are very short of water. The available water resources per capita are unevenly distributed. Of the available water resources, the freshwater resources are even more scarce. Moreover, this problem has a tendency to get worse year by year [13,14]. Due to the shortage of freshwater resources in inland and offshore areas, it is sometimes necessary to prepare fracturing fluids directly with high-salinity water (seawater or formation water) or oilfield wastewater [15]. Therefore, fracturing fluids with high salt-tolerance is the key to ensuring the success of unconventional oil and gas exploration and development. The so-called unconventional oil and gas refers to a type of oil and gas system whose reservoir, fluid characteristics and accumulation mechanism are different from those of conventional oil and gas system, and which are difficult to explore or effectively exploit by conventional exploration and development methods. For unconventional oil and gas reservoir such as tight sandstone gas, shale gas, shale oil, and coalbed methane, hydraulic fracturing is a very important method of exploration and development. Among them, zwitterionic hydrophobic associating polymer fracturing fluid is a very important research direction.

ZID_16_PM, a kind of zwitterionic hydrophobic associating polymer, is designed and synthesized in this paper. It has an anti-polyelectrolyte effect and can be used in high-salinity water. The design calls for using acrylamide as the main polymerization monomer, and the commonly used and widely available salt-tolerance anionic monomer 2-acrylamide-2-methylpropanesulfonic acid (AMPS) and cationic monomer methacrylatoethyl trimethyl ammonium chloride (DMC) are introduced into the polymer chain. Meanwhile, in order to further improve the salt-tolerance, a twin-tailed hydrophobic monomer DiC_16_AM, which has a strong hydrophobic effect and thickening performance, is also introduced into the polymer. Therefore, the study of zwitterionic hydrophobic associating polymers with excellent temperature-tolerance and salt-tolerance is of great significance to the development of oil and gas resources.

## 2. Experimental

### 2.1. Materials

Materials include ethanol, acetonitrile, dichloromethane, sodium hydroxide (NaOH), methacryloyl chloride, acrylamide (AM), sodium dodecyl sulfate (SDS), 2,2′-azobis (2-methylpropionamide) dihydrochloride (V50), sodium chloride (NaCl), potassium chloride (KCl), calcium chloride (CaCl_2_), and magnesium chloride (MgCl_2_), purchased from KeLong Chemical Co., Ltd. (Chengdu, China), and 1-Hexadecylamine, 1-bromohexadecane, 2-acrylamide-2-methylpropanesulfonic acid (AMPS), and methacrylatoethyl trimethyl ammonium chloride (DMC), obtained from Shanghai Aladdin BioChem Technology Co., Ltd. (Shanghai, China). All chemicals are chemical grade without further purification. Deionized water is prepared in the lab and used in all tests.

### 2.2. Synthesis of the Hydrophobic Associating Polymer ZID_16_PM

#### 2.2.1. Synthesis of the Twin-Tailed Hydrophobic Monomer DiC_16_AM

(1) Synthesis of the Intermediate DiC_16_

**Scheme 1:**
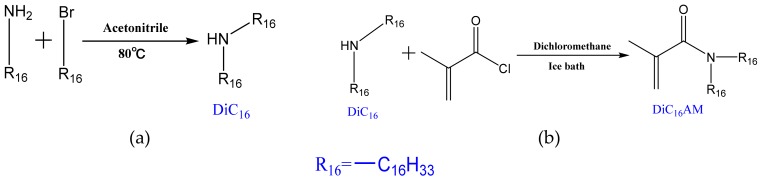
Synthesis of the (**a**) intermediate DiC_16_ and (**b**) twin-tailed hydrophobic monomer DiC_16_AM.

The intermediate DiC_16_ is synthesized from 1-hexadecylamine and 1-bromohexadecane. The 1-Hexadecylamine (12.073 g, 50 mmol) is dissolved in acetonitrile (250 mL) and poured into a three-necked flask (equipped with a condenser tube), and the three-necked flask is moved into an oil bath and stirred at 80 °C for 8 h. In the meanwhile, the 1-bromohexadecane (15.267 g, 50 mmol) is dissolved in acetonitrile (50 mL) and then added into the three-necked flask dropwise by constant pressure funnel. The synthetic route is shown in Scheme 1a. The solution in the three-necked flask is cooled to 25 °C for 2 h, at which point the product precipitates from the solution. A suction filter is used to remove the solution and obtain the dry product. Finally, the product is washed twice with dichloromethane and 6 mol/L NaOH solution and separated in a separatory funnel. The dichloromethane is removed on a rotary evaporator to produce the pure intermediate DiC_16_.

(2) Synthesis of the Twin-tailed Hydrophobic Monomer DiC_16_AM

The twin-tailed hydrophobic monomer DiC_16_AM is synthesized from the intermediate DiC_16_ and methacryloyl chloride. The intermediate DiC_16_ (18.6 g, 40 mmol), dichloromethane (100 mL) and 6 mol/L NaOH solution (50 mL) are added into a single-necked flask. The methacryloyl chloride (4.6 g, 44 mmol) is dissolved in dichloromethane (50 mL) and then added into the single-necked flask dropwise by constant pressure funnel. The single-necked flask is placed in an ice bath (2–5 °C) and stirred for 10 h, and the synthetic route is shown in Scheme 1b. Finally, the product is separated in a separatory funnel, washed twice with deionized water and once with concentrated NaCl solution, and dried over anhydrous magnesium sulfate. The solvent is removed on a rotary evaporator to yield a light yellow oil. And the light yellow oil is the twin-tailed hydrophobic monomer DiC_16_AM [16].

#### 2.2.2. Synthesis of the ZID_16_PM

**Scheme 2:**
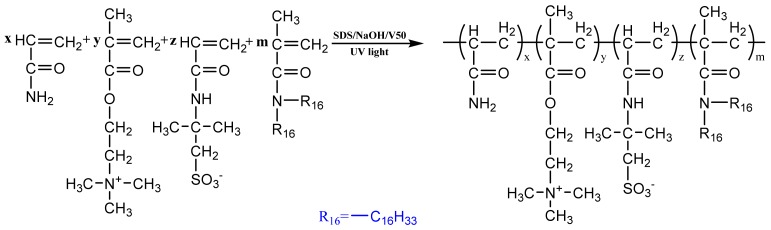
Synthesis of the ZID_16_PM.

The reaction monomers are AM, AMPS, DMC, and DiC_16_AM, and the molar ratio of AMPS and DMC is 1 to ensure equivalency of the positive and negative charges. The synthetic route is shown in Scheme 2. The reaction is conducted in a 100 mL beaker. The appropriate amounts of AM (9 g, 126.62 mmol), AMPS (2.99 g, 14.45 mmol), DMC (3 g, 14.45 mmol), and DiC_16_AM (0.15 g) are dissolved in deionized water, the total monomer concentration is maintained at 30 wt. %. And the mass ratio of additive SDS (1.5 g) to twin-tailed hydrophobic monomer is 10, and the pH is adjusted to seven using sodium hydroxide [17]. V50 solution (a solution of 1 wt. % V50 is prepared using deionized water) is added using a syringe, and the amount of V50 is 0.08 wt. % of the total mass of the solution. The beaker is then placed under a UV light fixture. Polymer colloid is obtained via illumination reaction after 8 h at 25 °C and cut into small pieces, then washed several times with ethanol. Finally, hydrophobic polymer product ZID_16_PM is acquired after vacuum drying and granulation. And we can get 13 g product ZID_16_PM.

### 2.3. Experimental Tests

The zwitterionic hydrophobic associating polymer, ZID_16_PM, is characterized by spectroscopy and is recorded in D_2_O at 400 MHz (Bruker AVANCE III HD 400, Bruker, Karlsruhe, Germany) at ambient temperature. The viscosity of ZID_16_PM with different concentrations (ZID_16_PM solutions are prepared using deionized water) and different salt concentrations (salt solutions are prepared using deionized water) is measured by a six-speed rotary viscometer (NDJ-95A, Shanghai, China) at 25 °C, through which the critical association concentration (CAC) of ZID_16_PM in aqueous solution and the effect of salt on the ZID_16_PM solutions are studied. The particle size of ZID_16_PM with different salts is determined by dynamic light scattering with a wide angle laser light scatterometer (Brookhaven, BI-200SM, Suffolk, NY, USA). A HAAKE MAR III RS 600 rheometer (Thermo Scientific, Munich, Germany) equipped with a high pressure sealed cell is employed to study rheological properties. According to the SY/T 6376-2008 general technical requirement of water-based fracturing fluids [18] and the SY/T 5107-2016 performance evaluation method [19], we conduct rheological measurements, proppant suspension tests, and core matrix permeability damage rate tests.

## 3. Results and Discussion

### 3.1. Structural Characterization

**Figure 2:**
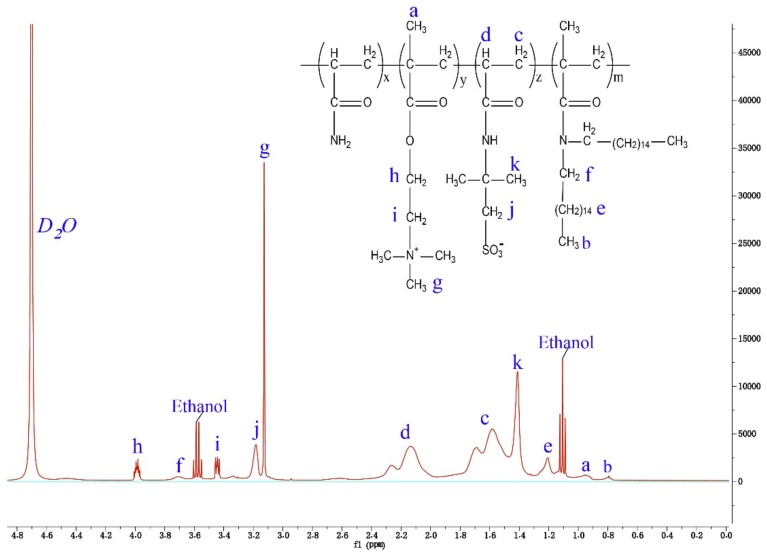
The ^1^H NMR of ZID_16_PM.

The structure of the hydrophobic polymer product, ZID_16_PM, is characterized by spectroscopy and confirmed by ^1^H NMR. Figure 2 shows the ^1^H NMR (400 MHz, D_2_O) spectrum of ZID_16_PM.

The resonances of protons are as follows: 4.70 (D_2_O) for the solvent peak; 0.793 ppm (b) for the methyl proton peak attached to the hydrophobic chain of DiC_16_AM; 0.932 ppm (a) for the methyl proton peak attached to the carbon atom of DMC and DiC_16_AM; 1.088–1.119 ppm and 3.537–3.610 ppm for ethanol solvent peaks; 1.170–1.243 ppm (e) for the methylene peak of DiC_16_AM; 1.361–1.471 ppm (k) for the methyl proton peak of AMPS; 1.480–1.782 ppm (c) and 2.010–2.334 ppm (d) for (–CH_2_–CH–, –CH_2_–C–) on the main chain; 3.128 ppm (g) for the methyl proton peak attached to the quaternary ammonium of DMC; 3.152–3.227 ppm (j) for the methylene peak attached to the sulfonic acid foundation group of AMPS; 3.418–3.469 ppm (i) for the methylene peak attached to the quaternary ammonium of DMC; 3.712 ppm (f) for the methylene peak attached to the tertiary amine of DiC_16_AM; 3.958–4.016 ppm (h) for the methylene peak attached to the oxygen atom of DMC. It is verified that the synthesized polymer is consistent with the designed ZID_16_PM.

### 3.2. Critical Association Concentration

**Figure 3:**
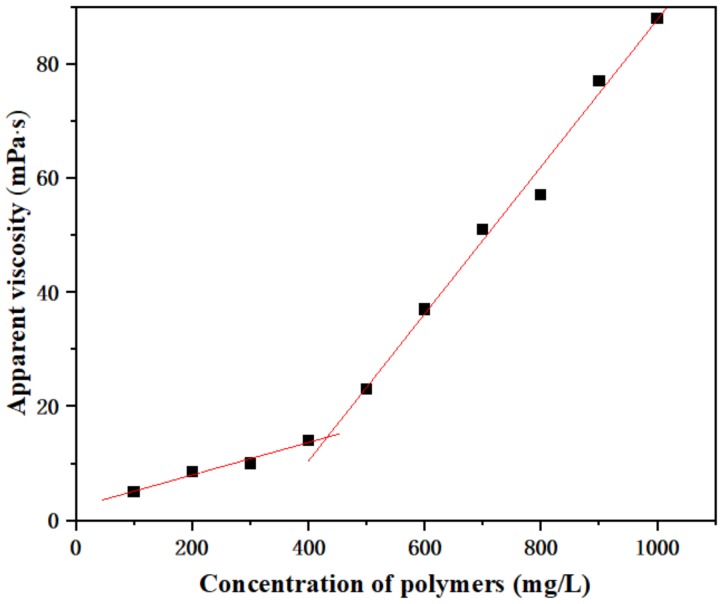
The critical association concentration (CAC) of ZID_16_PM.

The viscosity of ZID_16_PM at different concentrations in deionized water is measured by a six-speed rotary viscometer at 25 °C. Figure 3 depicts the plot of apparent viscosity versus the polymer concentrations showing that the polymer displays the remarkable thickening property of a directly proportional linear relationship. The apparent viscosity of the polymer solution increases with the increase of concentrations when a water-soluble polymer is hydrated. Due to a large dispersion of the polymer in low concentration solutions, hydrophobic chains of the polymer are mainly in intramolecular association. The number of polymer macromolecules in solution is scarce, and resulting in a scarce number of intramolecular association microregions formed by the hydrophobic interaction between the polymer solution’s hydrophobic groups. And the apparent viscosity increases slowly with increasing polymer concentrations. When the polymer concentration exceeds 500 mg/L, a large number of hydrophobic microdomains are exhibited, and the viscosity will increase sharply. This is because at this stage, the polymer solution’s macromolecular chains will become stretched, and hydrodynamic volume will increase significantly [20], and the formation of supramolecules via association and entanglement. The intersection point of the curve where the apparent viscosity increases sharply corresponds to the CAC of the ZID_16_PM. It is clear that polymers are associated at this concentration. The CAC is 433.63 mg/L for the ZID_16_PM, which is lower than common hydrophobic polymers (800–1500 mg/L) and shows a stronger hydrophobic effect. This is due to the introduction of the symmetrical twin tailed hydrophobic chains [21,22]. Symmetrical twin tailed hydrophobic chains have a stronger hydrophobic effect and thickening performance than single tailed hydrophobic chains, and they will further increase the binding forces due to the hydrophobic interaction [17]. When the concentration is greater than the CAC, the hydrophobic groups of ZID_16_PM are mainly in intermolecular association. Increasing polymer concentration leads to a decrease in the distance between polymer molecules. Therefore, the hydrophobic chains begin to have a greater intermolecular association, forming a dynamic physical cross-linking network. This results in a dramatic increase in fluid mechanical volume and viscosity. Polymer molecules can form supramolecular network structures through hydrophobic effect, electrostatic interaction, and hydrogen bonding, which increases the solution viscosity.

### 3.3. The Effect of Salt

#### 3.3.1. The Effect of Salt on Viscosity

**Figure 4:**
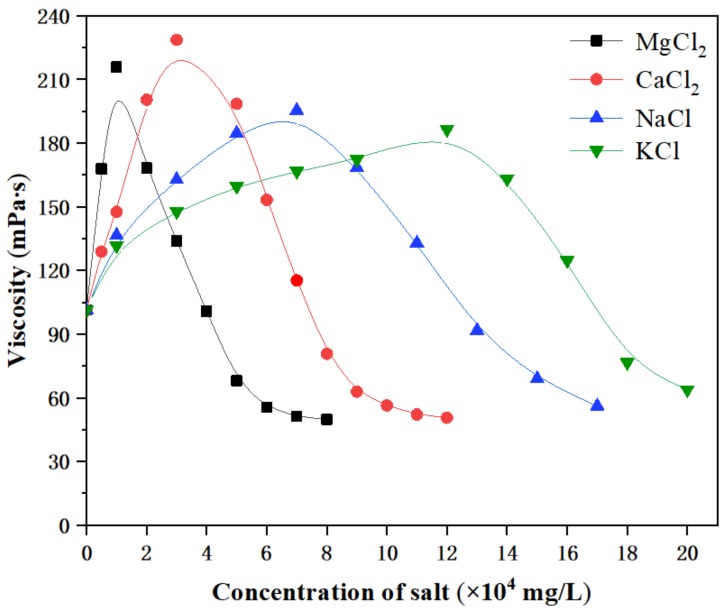
Effect of MgCl_2_, CaCl_2_, NaCl, and KCl concentrations on viscosity of a 0.3 wt. % ZID_16_PM aqueous solution.

We further evaluated the effect of the salt on the performance of ZID_16_PM by measuring the viscosity at different concentrations of MgCl_2_, CaCl_2_, NaCl, and KCl. Figure 4 shows the viscosity curves of 0.3 wt. % ZID_16_PM aqueous solution versus salt concentrations. The curves of different salts have similar trends with increasing salt concentration. The viscosities of MgCl_2_ and CaCl_2_ curves increase dramatically until the salt concentration reaches 1 × 10^4^ and 3 × 10^4^ mg/L, respectively, which is the salt concentration at which the viscosity peaks. With the further increase in salt concentration, the viscosities decrease sharply. In contrast, the viscosities of NaCl and KCl curves increase and decrease more stably and continue to reach peaks until the salt concentration reaches 7 × 10^4^ and 12 × 10^4^ mg/L, respectively. Finally, the viscosities of the four salt curves decrease to a stable value of about 50–60 mPa·s.

In the initial phase, due to the absence of inorganic salt ions, the polymer macromolecules mainly rely on the mutual attraction of their positive and negative charges and the intramolecular association microregions formed by the hydrophobic interaction between the polymer solution’s hydrophobic groups to produce viscosity. The hydrophobic chains are mainly in intermolecular association at this time, but the mutual attraction of positive and negative charges leads to the formation of internal salt bonds between polymer molecules, resulting in the curling of the polymer molecular main chains. Subsequent addition of a small number of inorganic salt ions, the viscosity of the polymer solution will have a rising stage. This is because the inorganic salt ions can break the internal salt bonds of the zwitterionic polyelectrolytes, which can extend the polymer macromolecules, and the hydrodynamic volume will increase significantly. As more hydrophobic microdomains are formed, the hydration shells of the polymer molecules also become thicker. From a macro-perspective, the solution viscosity has an upward trend at this stage. However, the number of positive and negative charges on the polymer molecules is limited, so that the viscosity of the polymer solution reaches a peak after a certain amount of inorganic salt is added. Subsequently, more inorganic salts are added to the polymer solution, and the excess inorganic salt ions dehydrate the polymer molecules, resulting in a thinning of the hydration shells of the polymer molecules [23]. Macroscopically, the viscosity of the solution tends to decrease. In addition, it can be seen from the Figure 4 that the four metal ions of K^+^, Ca^2+^, Na^+^, and Mg^2+^ have different effects on the viscosity of the polymer solution, which is attributed to the different hydration ionic radiuses of the four mental ions. The larger the hydrated ionic radius, the greater the strength of attraction between the inorganic salt ions and the water molecules, and the inorganic salt ions have a stronger dehydration ability. From a macroscopic point of view, the inorganic salt ions (Ca^2+^ and Mg^2+^) with a large hydrated ionic radius will cause the viscosity of the polymer solution to increase rapidly to a peak, and then the viscosity will decrease sharply to be stable.

#### 3.3.2. DLS Measurements

**Figure 5:**
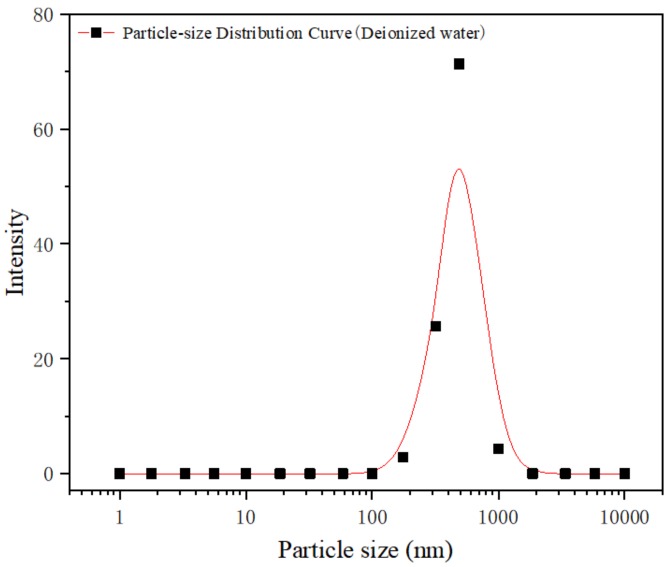
Particle size distribution of a 500 mg/L ZID_16_PM aqueous solution without salt.

**Figure 6:**
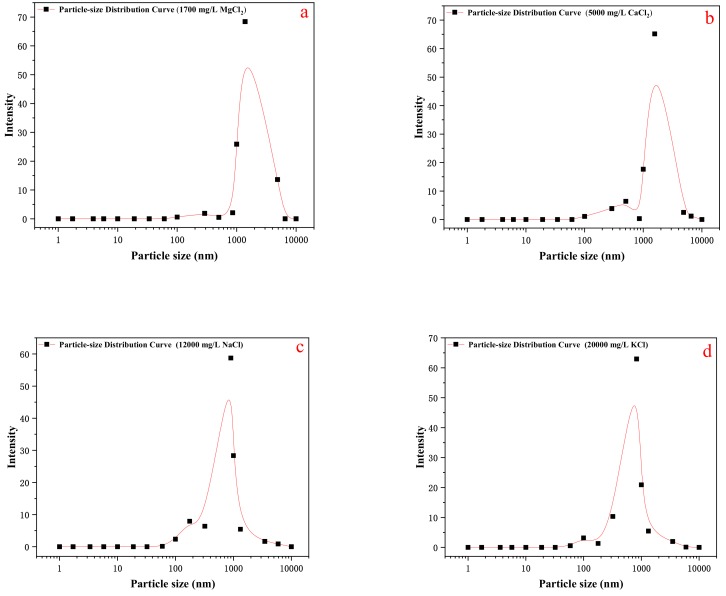
Particle size distribution of a 500 mg/L ZID_16_PM aqueous solution with various salt: 500 mg/L ZID_16_PM aqueous solutions with (**a**) 1700 mg/L MgCl_2_, (**b**) 5000 mg/L CaCl_2_, (**c**) 12,000 mg/L NaCl, and (**d**) 20,000 mg/L KCl.

The hydrodynamic radius (the hydrodynamic radius of a molecule refers to the radius of the moving particle corresponding to the measured diffusion coefficient of a different molecule [24]) of a polymer can represent its particle size and the size of polymer agglomerations in aqueous solutions. The 500 mg/L ZID_16_PM solutions used for DLS (dynamic light scattering) measurements are prepared by deionized water with the additions of MgCl_2_, CaCl_2_, NaCl, and KCl, respectively. The final analysis of data is performed by using CONTIN software, while the test temperature is 25 °C and the detection angle is 90°. According to the test results of the effect of salt on the viscosity of the ZID_16_PM in the laboratory, we choose the related salt concentration values based on the peak range of viscosity. We further conducted the particle size of ZID_16_PM with different salts as determined by dynamic light scattering [25,26]. The 500 mg/L ZID_16_PM aqueous solution and the 500 mg/L ZID_16_PM aqueous solutions with 1700 mg/L MgCl_2_, 5000 mg/L CaCl_2_, 12000 mg/L NaCl, and 20000 mg/L KCl are used to measure the particle sizes, respectively. The results are shown in Figure 5 and Figure 6. Under the condition of the 500 mg/L ZID_16_PM aqueous solution, the particle size is around 489.37 nm, and the particle sizes of the 500 mg/L ZID_16_PM aqueous solution with 1700 mg/L MgCl_2_, 5000 mg/L CaCl_2_, 12,000 mg/L NaCl, and 20,000 mg/L KCl are 1387.23, 1567.82, 901.47, and 828.49 nm, respectively. In the 500 mg/L ZID_16_PM aqueous solution, the polymer concentration is slightly greater than the CAC. Therefore, a large dispersion of the polymer in this concentrated solution, the anions, cations, and hydrophobic chains of the polymer, are associated mainly on the intramolecular level. The particle sizes of the polymer clearly increase after adding the four inorganic salts. This is due to the fact that inorganic salts can break the internal salt bonds of zwitterionic polyelectrolytes, which can extend the polymer molecules. It can also break the cross-linking between molecules [27]. This leads to an increase in polymer particle size. At the same time, the solution with divalent metal ions clearly has a larger particle size than the solution with monovalent metal ions. This indicates that divalent metal ions have stronger destructive ability to break the internal salt bonds of zwitterionic polyelectrolytes than monovalent metal ions.

In addition, it is worth emphasizing that the particle size distribution of the polymer ZID_16_PM in deionized water is relatively centralized. While the small second distribution occurs in these four inorganic salt ion solutions. This is due to the random and ruleless distribution of inorganic salt ions and polymer molecules in solution. And the intertwining of a small number of polymer molecules results in less influence of inorganic salt ions on them. However, the particle size distribution of most polymer molecules is still relatively centralized. The molecular dimensions measured at this time are mainly the size of the supramolecule [28], indicating that the inorganic salts can promote the interaction between polymer molecules.

In order to effectively weaken the influence of metal ions (such as K^+^, Ca^2+^, Na^+^, and Mg^2+^) in the modified polymer ZID_16_PM, AMPS, which is a salt-tolerant functional group; DMC, which is a cationic monomer; and DiC_16_AM, which has a strongly hydrophobic group, are introduced. AMPS is insensitive to the attack of external ions due to its strong anionic and water-soluble sulfonic acid group, so it is widely used as a salt-tolerant functional group in polymer design [29,30]. The purpose of introducing the cationic functional monomer DMC is to give polymer ZID_16_PM the same number of positive and negative charges simultaneously, so that the zwitterionic polymer has an anti-polyelectrolyte effect. A spatial network structure can be formed in the polymer aqueous solution due to the intermolecular hydrophobic association, which leads to the modified polymer ZID_16_PM with its strong salt-tolerance and thickening ability. However, high metal ion concentration results in the hydrophobic association with enhancement, and polymer molecule chains will become more tightly packed, which leads to a spatial structure change from a three-dimensional mesh space to a mass or sheet structure [31]. The tendency of polymer molecules to escape from inorganic salt solution increases, and the polymer will leave the liquid phase automatically when salting-out occurs, which causes the viscosity of polymer solutions to decrease [27].

**Figure 7:**
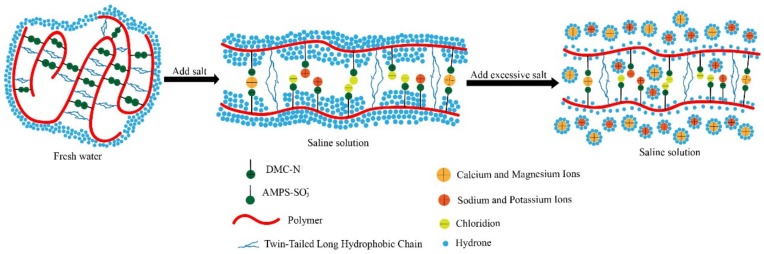
The effect mechanism of salt on ZID_16_PM aqueous solution.

A zwitterionic polymer is a polyelectrolyte by itself, which dissolves in deionized water, and ionization causes the counter-ion to diffuse from the polymer chain to the solvent region. As shown in Figure 7, because the zwitterionic hydrophobic associating polymer contains both cationic and anionic groups, the positive charges on the cationic groups and the negative charges on the anionic groups attract each other in fresh water, which causes the polymer molecules to curl up and, coupled with the mutual attraction of hydrophobic tail chains, these result in a fairly low initial polymer solution viscosity. With the addition of inorganic salts, which can break the internal salt bonds of zwitterionic polyelectrolytes and extend the polymer molecules, it can also break the cross-linking between molecules [27]. This is why the hydrodynamic radius of polymer molecules has increased. These result in the viscosity of the polymer solution rapidly rising until it reaches peak. When the concentration of inorganic salts is too high, the metal ions will dehydrate the hydrated cations and anions, and the polymer hydration shells will become thinner, which leads to a decrease of viscosity in the polymer solution. This is because the metal ions dissolved in the solution have a significant effect on the water molecules in the surrounding space. If the attractions of metal ions and water molecules are greater than the hydrogen bonds of water molecules, water molecules tend to accumulate near the metal ions to form hydration shells [32,33]. However, the strength of attractions depend on the hydration radiuses of the metal ions. The hydration radiuses of K^+^, Ca^2+^, Na^+^, and Mg^2+^ are 0.331, 0.412, 0.358, and 0.428 nm, respectively [34]. Therefore, Ca^2+^ and Mg^2+^ ions gather more water molecules from the hydration shells near the polymer molecule chains, which makes the hydration shells thinner and blocks the extension of polymer chains [23]. The hydration radius is larger, and the dehydration ability is stronger. This explains why ZID_16_PM has a wider salt tolerance to NaCl and KCl than it does to MgCl_2_ and CaCl_2,_ as shown in Figure 4.

### 3.4. Evaluation of the Fracturing Fluid Systems

#### 3.4.1. Rheological Measurement

With the broadening development of oil and gas fields, formation water increases year by year and needs to be treated. At the same time, the development of hydraulic fracturing technology with large displacement is required for site operation. Due to the shortage of freshwater resources in inland and offshore areas, it is sometimes necessary to directly prepare fracturing fluids with high-salinity water (seawater or formation water). Therefore, fracturing fluid with high salt-tolerance is the key factor for ensuring the success of unconventional oil and gas exploration and development. In this paper, the formation water of the West Sichuan Gas Field is directly prepared into fracturing fluid (Fluid-1) with a concentration of 0.3% ZID_16_PM, and the contents of K^+^, Ca^2+^, Na^+^, Mg^2+^, and Cl^−^ are 118.30, 2211.00, 14,492.00, 182.50, and 27,345.99 mg/L [35], respectively. The seawater of the Gulf of Mexico is also directly prepared into fracturing fluid (Fluid-2) with a concentration of 0.3% ZID_16_PM, and the contents of K^+^, Ca^2+^, Na^+^, Mg^2+^, and Cl^−^ are 470, 650, 11,000, 1220, and 19,700 mg/L [36], respectively. Related performance evaluations are conducted for each. 

**Figure 8:**
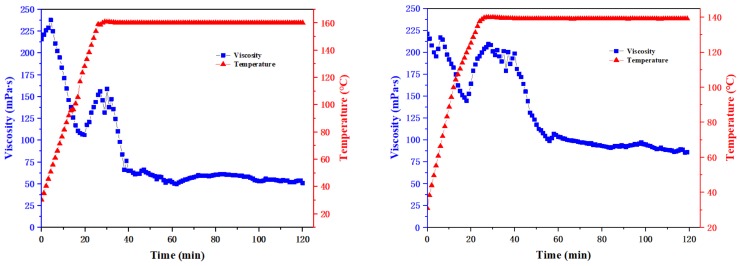
Rheological properties of the Fluid-1 at 140 and 160 °C.

**Figure 9:**
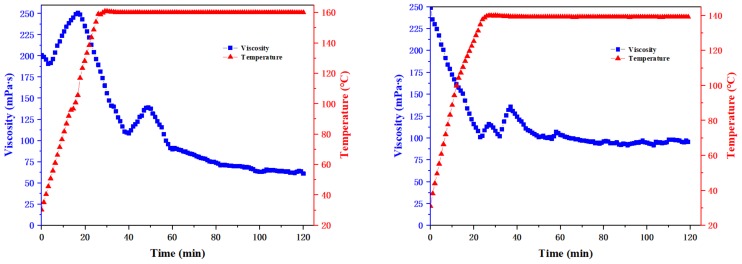
Rheological properties of the Fluid-2 at 140 and 160 °C.

We conduct temperature and continuous shearing resistance experiments at up to 140 and 160 °C at 170 s^−1^ for Fluid-1 and Fluid-2, respectively. Figure 8 and Figure 9 show the experimental results. The viscosity of Fluid-1 remains between 50 and 85 mPa·s, and the viscosity of Fluid-2 stays between 60 and 95 mPa·s, respectively, after 120 min of shearing. The SY/T 6376-2008 general technical requirements of fracturing fluids (viscosity is maintained above 30 mPa·s in the range of 120 to 180 °C) is used to evaluate the Fluid-1 and Fluid-2, both of which meet the standards [18]. Therefore, the test results indicate that ZID_16_PM can be applied as a fracturing fluid thickener and be beneficial to hydraulic fracturing field applications under high-salinity conditions.

**Figure 10:**
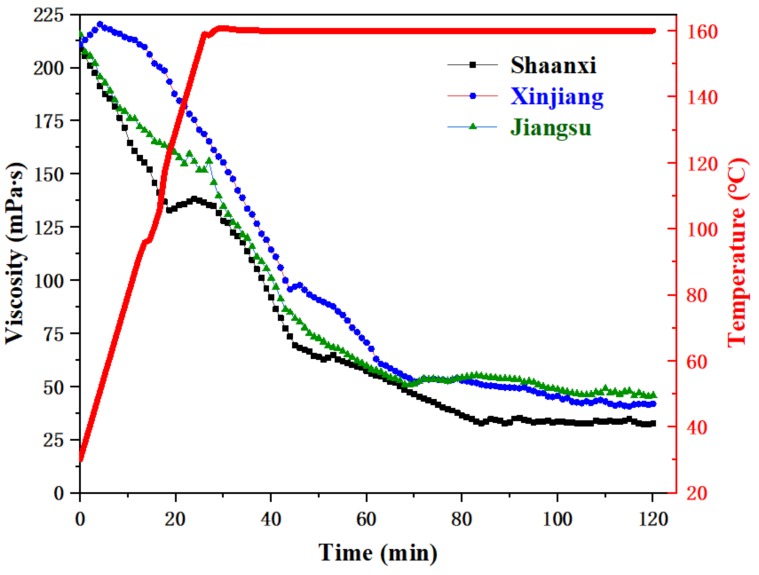
Rheological properties of the wastewater produced by an oilfield in Shaanxi, Xinjiang, and Jiangsu were prepared into fracturing fluids at 160 °C.

According to the above results, the wastewater produced by an oilfield in Shaanxi, Xinjiang and Jiangsu are prepared into fracturing fluids with the concentration of 0.3% ZID_16_PM, and we conduct relevant rheological experiments. Water quality analysis of the three oilfields is shown in the Table 1. And the Figure 10 shows that the viscosity of the wastewater produced by an oilfield in Shaanxi, Xinjiang and Jiangsu are prepared into fracturing fluids which can remain between 32, 42, 45 mPa·s, respectively, after 120 min of shearing at 160 °C. According to the SY/T 6376-2008 general technical requirements of fracturing fluids [18], these fracturing fluids all meet the requirements for hydraulic fracturing field applications. Therefore, these indicate that ZID_16_PM can also be applied as a fracturing fluid thickener to improve oil and gas recovery by using oilfield wastewater to prepare fracturing fluids.

#### 3.4.2. Proppant Suspension Test

**Figure 11:**
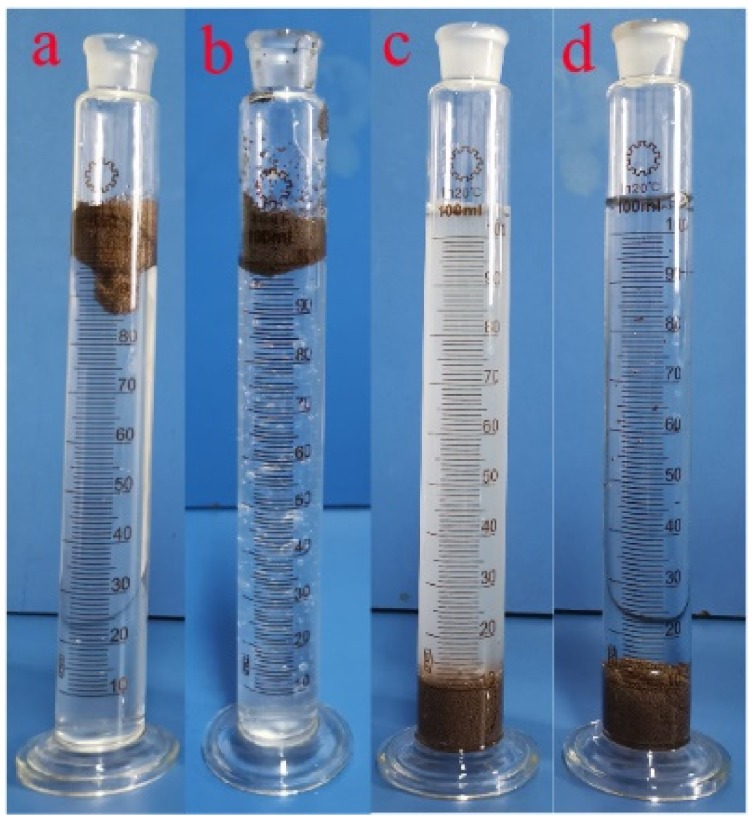
Result of proppant suspension measurements, (**a**) Fluid-1, (**b**) Fluid-2, (**c**) guar gum, and (**d**) linear glue.

A high-pressure pump set installed on the ground can continuously bring the fracturing fluids with proppant into the formation fractures, the cracks further extend forward, and finally the proppant will remain in the cracks. In order to ensure the smooth implementation of this process, the fracturing fluids are required to have an excellent proppant suspension capacity. Therefore, we use static proppant suspension tests to investigate the proppant suspension capacity of Fluid-1 and Fluid-2. At the same time, we introduce the proppant suspension ability of guar gum and linear glue for comparison. The proppant materials used for static proppant suspension measurements are ceramsite with the sizes ranging from 452 to 850 μm (20/40 mesh) and a density of 1.6 g/cm^3^, and the experimental processes are conducted in a 100 mL graduated cylinder at 90 °C by using the 10% (volume ratio) of the proppant concentration. As shown in Figure 11, the proppant suspension performances of the ZID_16_PM are far better than those of guar gum or common polymer (linear glue). In the heating conditions in a drying oven, proppant settlement occurs in all fracturing fluids. The settling velocity here refers to the distance that the particles fall in the measuring cylinder per unit time at a fixed temperature when settling in a stationary fluid, and the related parameters are listed in Table 2. The settling velocities for Fluid-1 and Fluid-2 are 1.45 × 10^−3^ and 1.98 × 10^−3^ mm/s, respectively. According to related research, we conduct proppant suspension tests for the guar gum and common polymer (linear glue) [37], and their fracturing fluids are 4.41 and 3.26 mm/s, respectively. The test results show that the polymer ZID_16_PM can be beneficial to hydraulic fracturing operations. Moreover, fracturing fluid can be prepared directly by using the formation water coming from the West Sichuan Gas Field and the seawater from the Gulf of Mexico.

#### 3.4.3. Core Matrix Permeability Damage Rate Test 

It is essential to reduce the damage induced by fracturing fluids to the oil and gas layers during the fracturing process [38]. Therefore, the SY/T 5107-2016 performance evaluation method [19] and the SY/T 6376-2008 general technical requirement of water-based fracturing fluids [18] are applied to test the core matrix permeability damage rate of Fluid-1 and Fluid-2. The core diameters of 25–25.4 mm and core lengths of 1–1.5 times the diameters used for forward and reverse flow experiments are selected to investigate the variation of core permeability. And kerosene is chosen as the flow medium. The related Equation 1, which is used to calculate the core matrix permeability damage rate [39], is as follows:

(1) $$\eta_{d}=\frac{K_{1}-K_{2}}{K_{1}}\times 100\%,$$

where *η_d_* is the core matrix permeability damage rate (%), *K*_1_ is the measured permeability before the fracturing fluids are injected into the cores (μm^2^), and *K*_2_ is the measured permeability after the fracturing fluids are injected into the cores (μm^2^).

The core matrix permeability damage rate of Fluid-1 and Fluid-2 are 13.77% and 11.62%, respectively. The results are listed in Table 3. Compared with the guar gum and common polymer (linear glue), Fluid-1 and Fluid-2 have lower core damage rates, and they all meet the SY/T 6376-2008 standard [18]. Therefore, the polymer ZID_16_PM is of benefit in hydraulic fracturing production operations with high-salinity water.

## 4. Conclusions

In this article, a novel zwitterionic hydrophobic associating polymer, ZID_16_PM, has been successfully and easily synthesized showing excellent performance as a thickener for fracturing fluid. The polymer ZID_16_PM has equivalent positive and negative charges and some hydrophobic monomers with twin-tailed long hydrophobic chains, so that it has an anti-polyelectrolyte effect and a stronger association force. Thus, ZID_16_PM also had many prominent advantages including excellent salt-tolerance, good rheological properties, outstanding proppant suspension, and minimal damage to the formation. We have conducted temperature and continuous shearing resistance experiments at up to 140 and 160 °C at 170 s^−1^ for Fluid-1 and Fluid-2. After 120 min of shearing, the viscosity of Fluid-1 remained in the range of 50 to 85 mPa·s, and the viscosity of Fluid-2 stayed within 60–95 mPa·s. And the wastewater produced by an oilfield in Shaanxi, Xinjiang, and Jiangsu have been prepared into fracturing fluids with a concentration of 0.3% ZID_16_PM, the viscosity of these fracturing fluids could remain 32, 42, and 45 mPa·s, respectively, after 120 min of shearing at 160 °C. Therefore, high-salinity water (seawater or formation water) or oilfield wastewater can be prepared for use directly as a fracturing fluid, which is of great significance to the areas where freshwater resources are scarce. Consequently, ZID_16_PM has profound importance for the development of oil and gas resources.

## Figures and Tables

**Table 1:** Water quality analysis of an oilfield in Shaanxi, Xinjiang, and Jiangsu.

Component	Content (mg/L)		
	Shanxi	Xinjiang	Jiangsu
K^+^, Na^+^	6542.685	4459	4817.01
Ca^2+^	7358.33	121.9	11.06
Mg^2+^	147.625	18.3	8.95
Fe	0.411	\	\
SO_4_^2−^	509	125.9	120.18
HCO_3_^−^	201.375	1804.4	3923.34
CO_3_^2−^	\	237.8	\
Cl^−^	23,269.985	5719.8	5102.14
TDS	38,030	11,585	13,982.68

**Table 2:** Evaluation results of the settling velocity of proppant.

Fracturing Fluid	Fluid-1	Fluid-2	Guar Gum	Linear Glue
Settling velocity (mm/s)	1.45 × 10^−3^	1.98 × 10^−3^	4.41	3.26

**Table 3:** Evaluation results of the core matrix permeability damage rate.

Fracturing Fluid	Permeability Before Damage (10^−3^ μm^2^)	Permeability After Damage (10^−3^ μm^2^)	Permeability Damage Rate (%)
Fluid-1	10.53	9.08	13.77
Fluid-2	10.76	9.51	11.62
Guar gum	11.83	8.44	28.66
Linear glue	11.56	9.27	19.81
SY/T 6376-2008	\	\	≤30
